# Exploring Chinese Consumers’ Online Purchase Intentions toward Certified Food Products during the COVID-19 Pandemic

**DOI:** 10.3390/foods10112729

**Published:** 2021-11-08

**Authors:** Xin Qi, Xu Tian, Angelika Ploeger

**Affiliations:** 1 Specialized Partnerships in Sustainable Food Systems and Food Sovereignty, Faculty of Organic Agricultural Sciences, University of Kassel, 37213 Witzenhausen, Germany; a.ploeger@uni-kassel.de; 2 College of Economics and Management, Academy of Global Food Economics and Policy, China Agricultural University, Beijing 100083, China; tianxu@cau.edu.cn

**Keywords:** certified food, online purchase intention, TPB, TAM, COVID-19, Chinese consumer

## Abstract

The outbreak of COVID-19 has significantly increased consumers’ demands for online groceries, as well as healthy, safe, and better-quality food products. In China, certified food products are commonly perceived as safe and good-quality products. Therefore, this study investigated potential factors that influenced Chinese consumers’ online shopping intentions toward certified food during the COVID-19 crisis. An integrated model was proposed by combining the technology acceptance model (TAM) and the theory of planned behaviour (TPB) with the impact of COVID-19 (IOC). The empirical results of structural equation modelling analysis with 491 usable responses revealed that the proposed model showed a good model fit and satisfactory explanatory power (R^2^ = 53%) regarding consumers’ certified online food shopping intentions during the pandemic. The path analysis demonstrated that attitude, perceived behavioural control, perceived usefulness (PU), and IOC significantly affected consumers’ online purchase intentions of certified food. PU and perceived ease of use (PEOU) were important drivers of attitudes, and PEOU significantly influenced PU. Moreover, the IOC was significantly related to most factors, except subjective norms. These findings can be useful for detecting changes in consumer behaviour, and providing suitable strategic implications for stakeholders in the Chinese certified food sector during the current and post-pandemic eras.

## 1. Introduction

One year after the World Health Organization (WHO) declared the outbreak of the novel coronavirus disease (COVID-19) as a pandemic [1], the virus is still rapidly spreading around the world, and affecting humankind at an unprecedented scale [2]. Although numerous measures have been imposed by governments to control the spread and transmission of the virus, the numbers of infections and deaths are still rising, with more than 225 million confirmed cases of COVID-19 globally in September 2021 [3]. The pandemic is far from being simply a health crisis—it is expected to cause a severe global economic disruption and affect all economic sectors, particularly the food sector [2]. Notably, it is evident that the pandemic has profoundly changed the food systems, as well as the way people purchase and consume their food [4,5,6,7,8].

A further consequence of the decreased accessibility to physical stores and individuals’ fears of being in proximity to others is the immediate increase in consumer demand for online groceries [9,10]. China is the world’s leading online retailer, with the largest digital buyer population in the world (i.e., more than 710 million people) [11]. Electronic commerce has skyrocketed in China, and has been predominant during the pandemic. During the early stage of the outbreak, China’s top three online grocery platforms (i.e., Alibaba’s online supermarket Hema, Miss Fresh, and JD.com) reported that orders were up 220%, 350%, and 470% year over year, respectively [12]. At the same time, increasing consumers have changed their shopping channels from offline and wet markets to online stores [13]. Thus, there is tremendous potential for the Chinese food industry to apply agile retailing, and scale up to online food shopping during and beyond the pandemic period. Moreover, global dietary and consumption patterns have undergone profound changes [14,15]. COVID-19 is likely to create a more sustainable, healthier era of consumption over the next ten years [16]. Some recent empirical studies revealed that certain consumers increasingly preferred to choose healthy, safe, and better-quality food products during the pandemic [17,18,19]. Products with certifications are commonly perceived as safe and good-quality products by consumers, since they promise higher standards, and provide consumers the ability to make safer and healthier food choices over the selection of standard products [20,21]. In this new trend, Chinese certified food products can satisfy the shifting needs and requirements of consumers obtained during the pandemic. Therefore, it is important for stakeholders in the Chinese certified labelled food industry to understand the online shopping behaviour of consumers, and anticipate the changing consumption pattern during this global crisis to maintain a competitive edge.

Hazard-free food, green food, organic food, and agro-product geographical indication products are the four major categories of certified food in China, and are collectively called “San Pin Yi Biao” [22]. Table 1 provides basic information and a comparison of the four kinds of Chinese food certifications. By the end of June 2020, there were 3090 agro-product geographical indications, 71,185 hazard-free foods, 38,545 green foods, and 4548 organic food certified products, as well as around 800 production bases in China [23]. Moreover, a recent investigation by Wang, Tao, and Chu [24] found that nearly 70% of consumers believe that Chinese certification labels can reassure and guarantee the safety and quality of their products. Food safety concerns are always an important consumption motive for Chinese consumers’ food choices [25]. Thus, expanding the development of certified food products can not only promote the sustainable development of the economy and environment, but also meet the current needs of Chinese consumers during the pandemic. Although previous studies have conducted research related to certified food products in China, they only focus on hazard-free food, green food, and organic food. These studies rarely incorporated agro-product geographical indication products into the investigation, therefore, their results are most likely biased. Thus, the present paper attempts to fill this knowledge gap by investigating these four types of certified food products in the Chinese context.

The COVID-19 pandemic has also disrupted retail, and increased electronic commerce because of its convenience, economic advantages, and flexibility [10]. Many recent studies stated that online shopping became the first choice of consumers during the pandemic period [27,28]. Nevertheless, there is limited scholarly research on Chinese consumers’ online shopping behaviours for purchasing certified labelled food products. In previous studies, the technology acceptance model (TAM), proposed by Davis [29], has been frequently employed to measure consumers’ online shopping behaviours [30,31]. Similarly, the theory of planned behaviour (TPB), developed by Ajzen [32], is another widely used conceptual framework to determine the driving forces of consumers’ food purchase intentions [33,34,35]. However, there are substantial criticisms of the TAM and TPB due to the parsimony and shortcomings of their standard frameworks [36,37]. In particular, many scholars have argued that the original components of the TAM and their relations are not powerful enough to predict an individual’s technology acceptance behaviour in different contexts [37,38]. Likewise, prior studies have reflected that a weakness of the TPB is its lack of explanatory power in the domain of information technology (IT) behaviours [39]. Given that the two models are complementary, some studies have combined the TAM and TPB frameworks to improve their effectiveness in explaining an individual’s behaviour toward using modern technology in different contexts [40,41,42]. However, to the best of our knowledge, no previous studies have integrated the TAM and TPB models to explain consumers’ online purchase behaviours for certified food products in the Chinese context, therefore, the effectiveness of this combination still needs to be examined. Furthermore, numerous recent studies have indicated that consumers’ shopping patterns have rapidly shifted toward online shopping during the pandemic [43,44], though these studies do not construct the specific factor of COVID-19 or investigate its impact on other determinants, thus, leaving a research gap that this paper attempts to fill.

This study aims to explore the appropriate framework for explaining Chinese consumers’ online purchase intentions toward certified food products during the ongoing COVID-19 pandemic. More specifically, this research provides a clear understanding of the factors influencing Chinese consumers’ online certified food purchase intentions by incorporating technology acceptance and behavioural analyses with the impact of COVID-19 (IOC). This study contributes to the understanding of Chinese consumers’ familiarity and purchasing experiences of different certified food products before and after the pandemic. Moreover, this is an initial study that practices the integrated TAM and TPB framework in the context of Chinese online certified food shopping. Finally, this study bridges the gap by identifying the association between the IOC and other endogenous variables of consumers’ online buying behaviours in the current pandemic. The results of this research are expected to provide timely insights into how COVID-19 shifts consumers’ technology acceptance and online purchase behaviours for certified food products during and after the pandemic. Through our findings, key stakeholders, such as online food retailers, marketers, associations, and policy makers, can develop and manage suitable strategies and initiatives to further promote certified food online consumption in China.

## 2. Theoretical Framework and Development of Hypotheses

### 2.1. Theoretical Framework

**Figure 1:**
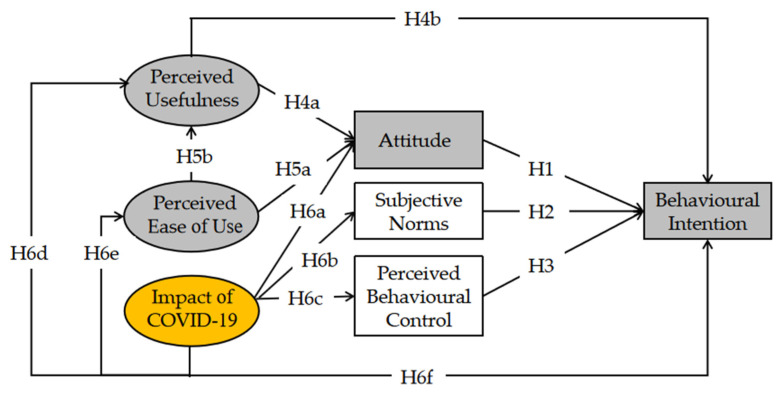
Research model. Note: The white and grey blocks are the constructs from the TPB framework; the grey blocks and grey ovals are the constructs in the TAM framework; and the yellow oval is a new pandemic variable added in the conceptual model.

This study develops an integrated framework by extracting variables from the TPB and TAM models, and incorporating the impact of COVID-19 to explain Chinese consumers’ online purchase intentions toward certified food during the pandemic time. Figure 1 represents the theoretical framework of this study. 

### 2.2. Development of Hypotheses 

#### 2.2.1. TPB

According to the TPB, an individual’s behavioural intention is guided by three determinants (i.e., attitude, subjective norms, and perceived behaviour control) [32].

- Attitude

According to Ajzen [32], attitudes capture an individual’s favourable or unfavourable assessment regarding the behaviour in question. Attitudes can be seen as constant predispositions toward subsequent behaviours. Therefore, they can be used to predict or anticipate such behaviour [45,46]. There is substantial empirical research available on understanding how consumers’ attitudes influence their behavioural intentions, which have observed a significant relation between the two [19,33,47]. For example, Lim and An [48] have recently studied healthy food consumption among 269 Korean consumers, and they identified that attitudes have made a significant contribution to the prediction of intention to purchase healthy foods. In terms of the studies on consumers’ online food purchase intentions, studies conducted by Liang and Lim [49], and Quevedo-Silva et al. [50] found that consumers’ attitudes toward buying food online have a significant positive impact on their behavioural intentions. Therefore, in line with the theory and the previously mentioned studies, we hypothesise the following:

**Hypothesis** **1** **(H1).** *Chinese consumers’ attitudes toward purchasing certified food through the internet are positively related to their online purchase intentions of certified food products*.

- Subjective norms

Subjective norms measure the perceived social influences/pressures to indulge or not to indulge in a given behaviour [32]. Subjective norms are a key determinant in social science studies because of their strong influence on individuals’ intentional behaviours [50]. Previous studies have shown that subjective norms can encourage the use of technologies and online purchases [51,52]. Consumers’ adoption of technology is affected by social forces related to their desires to comply with reference group norms [53], hence, subjective norms tend to guide group members’ intentions and behaviours [54]. For example, studies conducted by Akar [55] and Al-Swidi et al. [56] reported that subjective norms have a significant effect on purchase intentions online. Therefore, the above discussion leads to the second hypothesis of this study:

**Hypothesis** **2** **(H2).** *Subjective norms are positively related to Chinese consumers’ online purchase intentions toward certified food products*.

- Perceived behavioural control

Perceived behavioural control (PBC) concerns an individual’s perception of their ability to perform a behaviour of interest [32]. According to Thompson, Higgins, and Howell [57], PBC includes individuals’ perceptions of resources or knowledge to use the technology, and their abilities to perform the behaviour easily. The perception of users’ behavioural control is a salient factor of interactive technology adoption [51]. Studies in technology adoption conducted by Yang [52], and Kim et al. [54], have proven that PBC was the significant determinant of mobile shopping adoption in their investigations. According to Hsieh and Liao [58], and Al-Swidi et al. [56], PBC has a significant influence on an individual’s online shopping behaviour. In the present study, PBC is defined as consumers’ ability to purchase certified food from the internet when and how they want to. Accordingly, the following hypothesis can be proposed:

**Hypothesis** **3** **(H3).** *PBC is positively related to Chinese consumers’ online purchase intentions toward certified food products*.

#### 2.2.2. TAM

TAM posits two variables, namely, perceived usefulness (PU) and perceived ease of use (PEOU), as the primary triggers of individual attitudes toward technology-powered products/services that eventually induce behavioural intention [29].

- Perceived usefulness

According to Davis [29], perceived usefulness (PU) refers to the extent to which a person believes that using the technology or system will enhance their job performance. For the purpose of this research, we define PU as the consumers’ perception that purchasing certified food online will enhance their shopping experience and performance. Numerous empirical studies in the domain of electronic technology adoption have presented a significant relationship between PU and consumers’ attitudes and behavioural intentions. For instance, Chi [59] and Nassuora [60] found that PU exerted a significant, positive influence on the formation of consumers’ attitudes toward the use of mobile commerce. Studies from Moslehpour et al. [61], and Son et al. [62], asserted that PU has a positive effect on consumers’ intentions to use an internet application. In the field of online food consumption, Ramus and Asger Nielsen [63] found that the PU of online food shopping, with its convenience, wide range of products, and time savings, is one of the most significant factors driving consumers’ intentions toward online food shopping. Hence, the following hypotheses are proposed:

**Hypothesis** **4a** **(H4a).** *Chinese consumers’ PU of online certified food shopping positively influences consumers’ attitudes toward purchasing certified food through the internet*.

**Hypothesis** **4b** **(H4b).** *Chinese consumers’ PU of online certified food shopping has a positive impact on their online purchase intentions toward certified food products*.

- Perceived ease of use

Perceived ease of use (PEOU) is defined as “the degree to which a person believes that using a particular system would be free of effort” [29]. If consumers perceive that information technology is easier to use, they are more likely to accept it [29]. Studies in the area of food and technology have empirically demonstrated a significant association between PEOU and attitude, such as the studies from Nguyen et al. [64], and Kim and Woo [65]. Notably, Nguyen et al. [64] examined various factors affecting Vietnamese consumers’ acceptance of online food shopping, and reported that PEOU is the strongest predictor of attitudes. Moreover, based on the TAM, PEOU affects PU. Previous empirical studies have confirmed that PEOU positively affects PU at a significant level [59,64]. The easier consumers perceive using the internet to buy products online to be, the more useful online shopping is felt to be. Along with the previously conducted studies, the following hypotheses are proposed:

**Hypothesis** **5a** **(H5a).** *Consumers’ PEOU of online certified food shopping positively influences consumers’ attitudes toward purchasing certified food through the internet*.

**Hypothesis** **5b** **(H5b).** *Consumers’ PEOU of online certified food shopping has a positive impact on the PU of online certified food shopping*.

#### 2.2.3. The Impact of COVID-19

The global coronavirus pandemic has caused drastic changes in the structure of people’s daily routines around the world, including the way people buy and consume food [66]. Although it cannot determine whether consumers’ behavioural changes are permanent or temporary, the EIT-Food survey among 5000 consumers has reported that more than one-third believed their behaviour would be changed permanently after the pandemic [67]. The TPB has been widely used to explain consumers’ behaviours in different contexts [68,69]. Some recent studies have applied the TPB to investigate the impact of COVID-19 (IOC) on individuals’ changing behaviours. For example, Qi and Ploeger [19] measured the IOC on Chinese consumers’ purchase intentions toward green food based on the TPB framework, and demonstrated its significant effect on intentions. Nevertheless, their study merely examined the relationship between the IOC and behavioural intentions, and did not measure the association between the IOC and other constructs in the TPB model. Therefore, the present study responds to their calls to further investigate the relationship between the IOC and other salient factors. Recent studies have reported that individuals’ online shopping attitudes have shifted to different degrees during the pandemic [43,44,70]. Meanwhile, the IOC increased the uncertainty for consumers, which may encourage several adaptive behaviours, such as eating a healthy diet [71]. Consequently, the preference for certified food will increase, as they are commonly perceived as safe and healthy food. Thus, the IOC can promote positive attitudes towards online certified food shopping. In addition, people are more likely to follow others’ attitudes and behaviours when exposed to risk events such as SARS [72,73]. Notably, the recent study from Yang et al. [74] found that the pandemic has led people to be more willing to obey social norms, and follow collective behaviours in the consumption trends during the COVID-19 crisis. Therefore, the IOC should be positively associated with consumers’ subjective norms. Moreover, some studies have reported that individuals’ concerns about product shortages, high prices, and social distancing have increased due to COVID-19, which can impact their PBC [75,76]. Furthermore, the demand for online food shopping has dramatically grown due to the lockdown polices during the pandemic. Consumers decreased their frequency of market shopping, and switched to online shopping. Companies and retailers have put much effort into building, improving, and promoting their online offerings and services to cope with the increased demand, so consumers’ perceptions about using online shopping are correspondingly changing [9]. One study among Vietnamese consumers reported that the COVID-19 pandemic has affected consumers’ perceived benefits and usefulness of online shopping [77]. Consequently, we examine the IOC variable, and investigate its influences on each variable in the integrated model when facing the switch of customers’ behaviour in online shopping of certified food products. Accordingly, the following hypotheses arise:

**Hypothesis** **6a** **(H6a).** *The IOC is positively related to consumers’ attitudes toward online certified food shopping*.

**Hypothesis** **6b** **(H6b).** *The IOC is positively related to consumers’ subjective norms*.

**Hypothesis** **6c** **(H6c).** *The IOC is positively related to consumers’ PBC*.

**Hypothesis** **6d** **(H6d).** *The IOC is positively related to consumers’ PU of online certified food shopping*.

**Hypothesis** **6e** **(H6e).** *The IOC is positively related to consumers’ PEOU of online certified food shopping*.

**Hypothesis** **6f** **(H6f).** *The IOC is positively related to consumers’ online purchase intentions of certified food*.

## 3. Methodology

### 3.1. Data Collection

This study employed a quantitative design, and was conducted from June to July 2021 in China by means of an online survey. Given that our study aims to investigate individuals’ online shopping behaviour, internet access is essential, and the online survey approach is reasonable. Data were collected using a questionnaire survey platform (i.e., www.wenjuan.com; accessed on 1 June 2021). After a brief pilot test involving 20 consumers, the former questionnaire was adjusted and refined to improve comprehension and readability. The access link was distributed via WeChat, i.e., the most popular mobile messaging application in China, with more than 1 billion monthly active users. The target group of this survey included consumers older than 20, as this group accounts for the majority of Chinese consumers purchasing premium and green products [78]. Therefore, a filter question regarding the participant’s age was asked at the beginning of the questionnaire. The survey continued only if the age met the requirement. Altogether, 578 people took part in our investigation. After deleting incomplete and unreliable cases (e.g., missing answers and straight-line answer patterns), 491 respondents formed the final valid sample, which was used as the research dataset in our study (response rate = 84.95%). According to Kline [79], the minimum sample size for the empirical investigation is 10 cases per parameter. With 19 measurement items in our research, a minimum of 190 responses was required to examine the conceptual framework. Accordingly, under the aforementioned guidelines, our sample size of 491 valid responses was considered acceptable and statistically sufficient for this investigation.

### 3.2. Measures

The survey measurements were generated from the existing literature, and modified for this research. All measurements were back-translated by two native speakers to confirm the correct content and meanings. The questionnaire primary consisted of three sections. The first section contained questions in terms of respondents’ familiarity and their online shopping experience of different Chinese certified food products. The second section included questions in terms of behavioural attitudes, subjective norms, PBC, PU, PEOU, IOC, and online shopping intentions toward certified food. All measures of these variables were operationalised through a 7-point Likert scale, ranging from 1 (“strongly disagree”) to 7 (“strongly agree”). The questionnaire items and their sources of adoption are illustrated in Table 2. The third section comprised five items asking demographic characteristics.

### 3.3. Data Analysis

Data analyses were performed using Statistical Package for Social Science (SPSS) version 24 and Analysis of Moment Structure (AMOS) Version 24 (IBM Corp, New York, NY, USA). SPSS was used for descriptive analyses to analyse the characteristics of participants, and visualise the responses received. Then, AMOS was applied to test structural equation modelling (SEM) analysis through a two-stage procedure. In the first step, a confirmatory factor analysis (CFA) was conducted to evaluate the reliability and validity of the measurement model. In the second step, the full structural model was measured to evaluate the model fit and the hypothesised relationships, with the help of standardised regression coefficients (β), *t*-values, and *p*-values.

## 4. Results

### 4.1. Profile of the Respondents

Table 3 presents general information regarding the sample’s demographic features. The final sample in our study was highly educated (45.8% of respondents had a university degree or above), and contained more women (59.3%) than men. Participants’ ages ranged from 20 to 75 years, with a mean of M = 38.63 (SD = 13.23). The majority of respondents were married with one child or more (41.5%), and 39.7% of them reported that their annual household income was above RMB 100,000.

### 4.2. Descriptive Statistics

The descriptive statistics of respondents’ familiarity and their experience purchasing different Chinese certified food products are presented in Table 4. Among the Chinese certified food labelled products, respondents were most familiar with green food, followed by organic food and hazard-free food products. The agro-product geographical indication food label was the least familiar label, with less than one-third of participants having previous knowledge of it. Moreover, respondents were asked about their online shopping experience of certified food before and during the pandemic. Before the pandemic outbreak, 28.9% of participants purchased organic food through the internet, followed by green food (25.7%), hazard-free food (5.1%), and agro-product geographical indication products (2.2%). During the COVID-19 pandemic, the online purchases of most certified food increased to some different extent, except for agro-product geographical indication products. 

### 4.3. Measurement Model: Reliability and Validity

The reliability and validity test results are shown in Table 5. The estimate of Cronbach’s ɑ ranged from 0.854 to 0.925, and was within the acceptable limit of 0.7 and higher [82]. Therefore, all of the internal consistency levels of each structure show satisfactory levels. Regarding the convergent validity, the value of C. R ranged from 0.859 to 0.926, which implies that all constructs met the acceptable criterion of 0.6 or higher [83]. The factor loading value for all items ranged from 0.713 to 0.948, which was greater than the acceptable limit of 0.6 [84]. The value of AVE ranged from 0.671 to 0.806, which maintained the criterion of above 0.5 [82]. Accordingly, convergent validity was established. The square root of the AVE of each construct was higher than the correlation between the constructs, which guarantees adequate discriminant validity. After CFA analysis, it could be summarised that the conceptual framework represents adequate reliability and validity (convergent and discriminant). Table 6 highlights the details of discriminant validity.

### 4.4. Structural Model: Goodness of Fit Statistics

Table 7 shows the calculated fit indices for measuring model fit. Structural analysis shows that the proposed theoretical framework represents a good model fit (χ^2^/df = 2.670, GFI = 0.927, TLI = 0.955, CFI = 0.964, IFI = 0.964, RMSEA = 0.058). All indices met the recommended criterion, therefore, it can be concluded that the model is acceptable. Notably, the proposed model has satisfactory explanatory power for behavioural intentions (R^2^ = 0.53), which indicates that the present model can explain 53% of the total variance in this study.

### 4.5. Hypothesis Testing

The proposed significant effects of hypothesised paths were tested within the proposed conceptual framework. The standardised parameter estimates, *t*-values, significance levels, and the results of hypotheses are shown in Table 8. Of the 13 hypotheses, 11 were supported by structural model tests, which confirmed the proposed direction of significant effects, but two were not supported. 

Regarding the variables derived from the TPB framework, the results found that Chinese consumers’ attitudes toward online purchases of certified food (β = 0.280, t = 5.124, *p* < 0.001) and PBC (β = 0.341, t = 6.283, *p* < 0.001) significantly and positively affect their online shopping intentions of certified food, which supports H1 and H3. H2 proposed that consumers’ subjective norms would have a significant and positive effect on their behavioural intentions. Contrary to expectations, our results presented an insignificant effect on consumers’ online shopping intentions of certified food products (β = 0.068, t = 1.753, *p* > 0.05), thus, H2 is not supported. In terms of constructs stemming from the TAM framework, both consumers’ PU and PEOU of online certified food shopping significantly influence consumers’ attitudes toward purchasing certified food through the internet, which supports H4a (β = 0.427, t = 8.040, *p* < 0.001) and H5a (β = 0.205, t = 4.163, *p* < 0.001), respectively. H4b predicted a significant and positive relationship between consumers’ PU and their online shopping intentions of certified food products, and this relationship was confirmed by the results (β = 0.186, t = 3.112, *p* < 0.01). Therefore, H4b is supported. As proposed in H5b, consumers’ PEOU of online certified food shopping has a significant and positive effect on their PU of purchasing certified food through the internet (β = 0.338, t = 7.861, *p* < 0.001), so H5b is supported. Regarding the role of IOC, there were significant and positive associations between IOC and consumers’ attitudes (β = 0.190, t = 4.608, *p* < 0.001), PBC (β = 0.425, t = 8.579, *p* < 0.001), PU (β = 0.336, t = 6.963, *p* < 0.001), PEOU (β = 0.324, t = 6.662, *p* < 0.001), and behavioural intention (β = 0.109, t = 2.376, *p* < 0.05). Thus, H6a, H6c, H6d, H6e, and H6f are supported, accordingly. However, the analysis results showed an insignificant effect of the IOC on subjective norms (β = 0.023, t = 0.413, *p* > 0.05). Therefore, H6b is not supported. Table 8 shows detailed results.

## 5. Discussion

The COVID-19 pandemic was a major disruption and evidenced a certain amount of behavioural changes in individuals’ food consumption, resulting in the increased demands of online shopping for healthy, safe, and better-quality food products. Certification differentiates products, promising higher standards, and provides a channel for consumers to choose safer and healthier products than standard ones [24]. Therefore, the present study focuses on investigating Chinese consumers’ online shopping intentions of certified food during the COVID-19 pandemic. An integrated framework that extracted variables from the TPB and TAM models was proposed, and measured the impact of the pandemic on the online shopping behaviour for certified food products. SEM analytical results verified the applicability of the proposed model, and affirmed a set of causal links among the different factors of Chinese consumers’ online shopping intentions of certified food products during the pandemic.

Our descriptive findings regarding consumers’ familiarity and their experience of purchasing different Chinese certified food products confirmed some previously reported findings, but we also generated several new observations. For example, Liu et al. [26] reviewed Chinese consumers’ attitudes and behaviours toward certified food, and found that green food has the highest consumer awareness, followed by organic and hazard-free food products. This study reached a similar conclusion regarding the high awareness of green and organic food. However, our findings showed that most of our participants had not previously noticed the agro-product geographical indication label (72.3%), which reflected its serious information asymmetry between consumers’ cognition and the Chinese domestic certified food market. Moreover, our investigation indicated that the pandemic has increased the purchases of most certified food products. This finding is not in line with previous findings that reported the negative impact of COVID-19 on consumers’ green food purchases [85]. These conflicting results could be due to consumers’ shifting attitudes and behaviours during different pandemic stages. Qi et al. [85] conducted an investigation during the initial outbreak stage of COVID-19, and most consumers reduced their green food purchases due to unavailability issues, price issues, and panic issues. However, with numerous measures being implemented to curb the pandemic and solve the emerging problems of food supply chains, several recent studies reported that the pandemic has a positive impact on healthier eating and consumption habits [17,86]. 

For the effects of variables stemming from the TPB, PBC was found to have the greatest level of influence on their online certified food shopping intentions, which is in line with prior research by Liang and Lim [49]. This implies that improving individuals’ perceptions of the accessibility and convenience of purchasing certified food online plays an essential role in stimulating their intentions. Therefore, online certified food retailers should optimise the design of their platforms to meet consumers’ requirements, with respect to ease of use and control. In addition, attitude was also reported to significantly influence individuals’ intentions to purchase certified food products online in a positive way, which is consistent with the results of Liang and Lim [49], and Akar and Dalgic [87]. Surprisingly, consumers’ subjective norms presented an insignificant impact on their online purchase intentions, which were different results from recent online shopping studies, such as the investigations conducted by Bezirgani and Lachapelle [88], and Akar [55]. A possible explanation of this incongruence is due to the unstable predictive power of subjective norms in different contexts [36]. According to Venkatesh and Davis [38], user acceptance research examining the direct effect of subjective norms on an individual’s intention has yielded mixed results ranging from no significant effect to a significant effect. Our findings are also in line with other studies showing that not all important referents are perceived by individuals to influence their decisions [19,40]. The other inconsistent findings between our study and prior research are the different significant degrees of these three original factors. Previous studies reported that PBC was usually less effective than the other two constructs [87,89], whereas our study found that PBC was the most effective factor. This phenomenon could be due to consumers paying more attention to the accessibility and convenience of online shopping to address supply issues during the pandemic. Therefore, marketers should promote these benefits, such as saving their time, physical and mental effort, and energy to consumers to increase their intentions during the current COVID-19 pandemic. Meanwhile, online retailers should highlight the available benefits of their services, such as displaying the images of their popular certified food products and brands in the prominent place of their platforms.

With respect to the constructs from the TAM framework, PU and PEOU were found to have significantly positive effects on consumers’ attitudes toward online certified food shopping, which is consistent with the findings of previous studies [65,90]. These results suggest that effective marketing strategies and a well-functioning platform design for online shopping are essential for increasing consumers’ positive perceptions of buying certified food products online. In particular, companies should put effort into their online business to be easy to understand and easy to operate for customers, while still clearly displaying the values and quality of their certified food products. Moreover, our results found that PEOU significantly influenced PU in a positive way, which is in line with prior research by Nguyen et al. [64], and Kim and Woo [65]. This indicates that improving consumers’ PEOU can enhance their beliefs about the usefulness and effectiveness of online certified food shopping. Furthermore, PU was found to be a significant driver of consumers’ intentions to purchase certified food products online, which coincides with the earlier empirical findings from Ramus and Asger Nielsen [63]. This finding reveals that a positive PU leads to a greater likelihood for Chinese consumers to purchase online certified food. Thus, marketers should prioritise usefulness in their applications and services, such as emphasising the time savings, convenience, and usefulness of online shopping to improve consumers’ shopping performance, and create a satisfactory customer experience. 

In regard to the IOC, our results indicated that there were significant and positive associations between the IOC and consumers’ attitudes, PBC, PU, PEOU, and behavioural intentions. These findings indicate the necessity of incorporating the specific construct of the IOC when studying consumer shopping behaviours during the pandemic period. In particular, our findings revealed that the pandemic has shifted individuals’ online shopping behaviour and patterns, which is congruent with recent studies during the COVID-19 pandemic [88,91], and previous studies during other pandemics or crises [92,93]. Specifically, the IOC has greatly increased an individual’s attitude and intention toward purchasing certified food through the internet. This is possible because online shopping is commonly seen as the best optimal alternative during this crisis, which can meet individuals’ consuming demands, and prevent the risks of spreading the virus [92]. Thus, facing the rise in willingness and existing challenges during the pandemic period, the Chinese certified food industry should quickly adjust its production, supply, and marketing strategies to better respond to increased online demands. In addition, COVID-19 was also found to have a significant positive effect on consumers’ perceptions of control, ease of use, and usefulness of online shopping, which reflects that companies’ efforts to improve and facilitate their online offerings and services have been accepted by most customers during the pandemic. Therefore, enterprises should keep improving and upgrading their online platforms to address the changing online shopping needs at different pandemic stages. Interestingly, the IOC was found to have no significant association with subjective norms. One possible reason is the research object (i.e., online purchasing) and period (i.e., COVID-19) of our study, where consumers usually conduct those purchase-related activities at home, and reference groups can impose little influence. 

Finally, in terms of the overall performance of the proposed conceptual framework, our results indicated that the model presented a good model fit and exhibited good explanatory power, collectively accounting for 53% of the variance in Chinese consumers’ online shopping intentions toward certified food products during the pandemic crisis (R^2^ = 53%).

## 6. Conclusions

The diffusion of the COVID-19 pandemic has influenced consumers’ food shopping behaviours, which has led to increased online shopping and purchases of certified food products. Therefore, it is essential to explore the dynamics of consumers’ online certified food shopping behaviours during crisis periods. This study embraced a research model by integrating two existing models (i.e., TPB and TAM) and incorporating the effect of COVID-19 to better explain Chinese consumers’ online shopping intentions toward certified food products in the context of the current pandemic.

The conceptual framework and findings of empirical analyses highlight some theoretical and practical contributions. First, the present study expands previous consumer research on Chinese certified food products by investigating the four main types of Chinese certified food products. The findings reflected that they were commonly recognised and accepted among most Chinese consumers, and the demands of online shopping for certified food products increased during the pandemic period. Second, our investigation has suggested that TPB, as well as TAM, jointly affect individual behavioural intentions in online certified food shopping environments. The results strongly confirm the credibility of the TPB and TAM models’ assessments of individuals’ technology acceptance and behavioural performance in the different contexts. The study results reinforce existing evidence that factors such as attitude, PBC, PU, PEOU, and IOC have played significant roles in intentional processes of buying certified food online during the pandemic crisis. Third, this work is among the first attempts to explore the impact of COVID-19 on Chinese consumers’ online shopping behaviours of certified food products. Fourth, our study can potentially map a pathway for key stakeholders, such as online food retailers, website developers, marketers, and policy makers, to rethink strategies and tactics to further expand the Chinese certified food online market.

Although this study provides a better understanding of the key factors influencing Chinese consumers’ online certified food shopping intentions in the pandemic era, some limitations are worth mentioning for future research. First, this research is limited by measuring the underlying influence on consumers’ online shopping intentions, whereas the final actual online purchase behaviour is not measured. Since there exists inconsistency between intention and final behaviour, future studies can extend our framework to final online purchase behaviour to substantiate current research findings. Second, this research did not incorporate the important constructs of consumers’ familiarity, purchasing experiences, and trust into the theoretical model, thus, future research can integrate these factors, and examine their influences via empirical investigations. Third, the participants in our survey had higher education levels compared to the education level distribution of average Chinese consumers, which might reflect the notion that the population of online shopping buyers in general is well educated in China. Further research can conduct segmented investigations of targeted buyers, especially for consumers with university degrees or a higher level of education. Fourth, since individual shopping and consumption behaviours may shift during the different pandemic stages, some other unforeseen factors contributing to the diversities at a more aggregate level may have been overlooked and changed. Therefore, future research can follow consumers’ shifting demands and behaviours throughout other stages in the pandemic, and investigate other perceived determinants that hinder consumers from adopting online certified shopping (e.g., perceived barriers and perceived risks).

## Figures and Tables

**Table 1:** Comparison of agro-product geographical indications, hazard-free food, green food, and organic food certifications.

Certification	Agro-Product Geographical Indication	Hazard-Free Food	Green Food	Organic Food
Logo	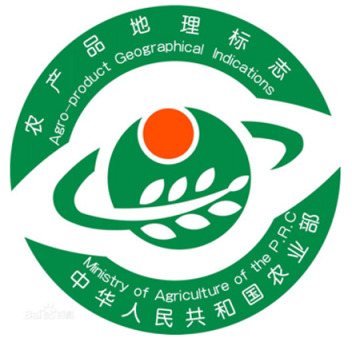	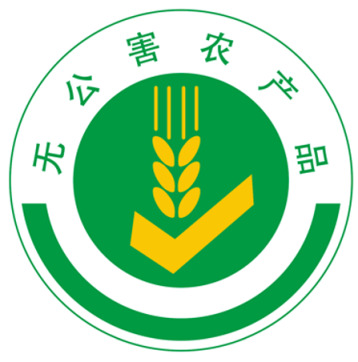	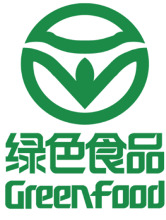	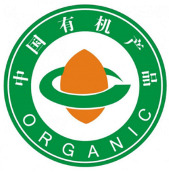
Definition	Agricultural products coming from the specified geographical region, whose quality and characteristics mainly depend on the natural ecological environment and historical and cultural factors, and who are named based on their regions.	Non-processed or initially processed edible agricultural products whose production environment, process, and quality meet the requirements of the relevant national standards and norms.	Safe and premium edible agricultural products and related processed products grown in an ecologically sound environment, which are produced according to the green food production standard, adopt the whole-some quality control, and obtain the right to use the “green food” logo.	Agricultural products and related processed products, which are organically produced and processed; no chemical synthetic pesticides, veterinary drugs, feed additives, etc., are used; no genetic engineering techniques are used; international or national organic requirements and standards are met; and certification from certified authorities is provided.
Requirements	No requirements of the safe index	Pesticide residue, heavy metal, harmful microorganisms, and other hygienic index meet the standard of hazard-free food products	Controlled and limited use of artificially synthesised fertiliser, pesticides, hormones, and other synthetics	No use of artificially synthesised fertiliser, pesticide, hormones, or other synthetics
Certificate authority	Centre for Agri-food Quality and Safety	Centre for Agri-food Quality and Safety	China Green Food Development Centre	Authorities approved certification
Validity of certification	Long term	3 years	3 years	1 year
Product positioning	Product-unique features and meeting specific consumption requirements	Guarantee of basic safety and meeting public consumption requirements	Domestic large- and medium-sized cities and the international market	The high-end domestic consumption and international market

Source: Own elaboration from [21,22,26].

**Table 2:** Questionnaire items and their source of adoption.

Variables	Items	Measurement Items	Adopted from
Behavioural Intention (PI)	BI1	I intend to use online shopping for certified food purchasing shortly.	Nguyen et al. [64]
	BI2	I predict I will regularly use online shopping for certified food purchasing in the future.	
Attitude (AT)	AT1	Buying certified food online is a good idea.	Lin [80]
	AT2	Buying certified food online is a wise idea.	
	AT3	I like to purchase certified food through the internet.	
Subjective Norms (SN)	SN1	Most people important to me think that I should use online shopping for purchasing certified food.	Han et al. [81]
	SN2	Most people I value would use online shopping for purchasing certified food rather than other approaches.	
Perceived Behavioural Control (PBC)	PBC1	If I want to, I can easily buy certified food online.	Han et al. [81]
	PBC2	I have resources, time, or opportunities to purchase certified food online.	
	PBC3	To buy or not to buy certified food online is entirely up to me.	
Perceived Usefulness (PU)	PU1	Certified food online purchasing enables me to save my time.	Nguyen et al. [64]
	PU2	Using online shopping for certified food makes it more effective to do my shopping.	
	PU3	Using online shopping for certified food facilitates comparative shopping.	
Perceived Ease of Use (PEOU)	PEOU1	Learning to operate online food shopping is easy for me.	Nguyen et al. [64]
	PEOU2	I find it easy to become skilled at purchasing certified food online.	
	PEOU3	It is easy to order certified food online.	
Impact of COVID-19 (IOC)	IOC1	I feel the coronavirus pandemic has affected me personally.	Meixner and Katt [18]
	IOC2	I feel the coronavirus pandemic will change my consumption pattern.	
	IOC3	I feel the coronavirus pandemic will change society.	

**Table 3:** Demographic characteristics of the samples (*n* = 491).

Demographic Variables		Frequency	Percent (%)
Gender	Male	200	40.7
	Female	291	59.3
Age	20–30	177	36.1
	31–40	107	21.8
	41–50	88	17.9
	51–60	82	16.6
	>60	37	7.6
Education	Junior school or below	103	21.0
	High school or technical secondary school	163	33.2
	University or above	225	45.8
Marriage	Married with a child or children	204	41.5
	Married	101	20.6
	Single	160	32.6
	Other	26	5.3
Household income (per year)	<RMB 50,000	47	9.5
	RMB 50,000–80,000	115	23.5
	RMB 80,000–100,000	134	27.3
	>RMB 100,000	195	39.7

**Table 4:** Respondents’ familiarity and their online purchase experience of different Chinese certified food products.

	Agro-Product Geographical Indication (%)	Hazard-Free Food (%)	Green Food (%)	Organic Food (%)
Respondents did not know it before	72.3	23.2	6.1	7.8
Respondents did know it before	27.7	76.8	93.9	92.2
Respondents have purchased online before the pandemic	2.2	5.1	25.7	28.9
Respondents have purchased online during pandemic period	1.6	5.3	36.6	34.2

**Table 5:** Measurement model: reliability and validity.

Constructs	Factor Loadings	C. R	SMC	AVE	Cronbach’s α	√AVE
BI		0.885		0.794	0.887	0.891
BI1	0.885		0.784			
BI2	0.897		0.805			
AT		0.859		0.671	0.856	0.819
AT1	0.724		0.524			
AT2	0.863		0.746			
AT3	0.863		0.744			
PBC		0.868		0.690	0.859	0.831
PBC1	0.713		0.508			
PBC2	0.919		0.844			
PBC3	0.846		0.716			
SN		0.862		0.758	0.854	0.871
SN1	0.948		0.898			
SN2	0.786		0.618			
PU		0.873		0.697	0.880	0.835
PU1	0.883		0.779			
PU2	0.862		0.744			
PU3	0.754		0.568			
PEOU		0.926		0.806	0.925	0.898
PEOU1	0.901		0.812			
PEOU2	0.908		0.825			
PEOU3	0.884		0.781			
IOC		0.887		0.724	0.890	0.851
IOC1	0.876		0.768			
IOC2	0.839		0.703			
IOC3	0.837		0.701			

Note: BI, behavioural intention; AT, attitude; PBC, perceived behavioural control; SN, subjective norms; PU, perceived usefulness; PEOU, perceived ease of use; IOC, impact of COVID-19; SMC, squared multiple correlation (i.e., squared value of correlation between the constructs); C. R, composite reliability; √AVE, square root of average variance extracted.

**Table 6:** Correlation matrix for discriminant validity.

	IOC	PEOU	PU	PBC	AT	SN	BI
IOC	**0.851**						
PEOU	0.324	**0.898**					
PU	0.446	0.447	**0.835**				
PBC	0.425	0.138	0.574	**0.831**			
AT	0.447	0.458	0.603	0.354	**0.831**		
SN	0.023	0.007	0.010	0.010	0.010	**0.871**	
BI	0.463	0.294	0.600	0.594	0.562	0.079	**0.891**

Note: BI, behavioural intention; AT, attitude; PBC, perceived behavioural control; SN, subjective norms; PU, perceived usefulness; PEOU, perceived ease of use; IOC, impact of COVID-19; the bold diagonal values represent the square root of AVE.

**Table 7:** Summary of fit indices from confirmatory factor analysis.

Fit Indices	Model	Recommended Value	Results
χ^2^/df	2.670	>1 and <5 *	Satisfactory
GFI	0.927	≥0.9 *	Satisfactory
TLI	0.955	≥0.9 *	Satisfactory
IFI	0.964	≥0.9 *	Satisfactory
CFI	0.964	≥0.9 *	Satisfactory
RMSEA	0.058	≤0.08 *	Satisfactory
R^2^	0.53		

Note: * Source from Bagozzi and Yi [83]; GFI, goodness-of-fit index; NFI, normative fit index; TLI, Tucker-Lewis index; CFI, comparative fit index; IFI, incremental fit index; RMSEA, root mean square error approximation.

**Table 8:** Hypotheses test results.

Hypothesised Path	Standardised Path Coefficients	*t*-Value	Results
H1: AT → BI	0.280	5.124 ***	Supported
H2: SN → BI	0.068	1.753	Not supported
H3: PBC → BI	0.341	6.283 ***	Supported
H4a: PU → AT	0.427	8.040 ***	Supported
H4b: PU → BI	0.186	3.112 **	Supported
H5a: PEOU → AT	0.205	4.163 ***	Supported
H5b: PEOU → PU	0.338	7.861 ***	Supported
H6a: IOC → AT	0.190	4.608 ***	Supported
H6b: IOC → SN	0.023	0.413	Not supported
H6c: IOC → PBC	0.425	8.579 ***	Supported
H6d: IOC → PU	0.336	6.963 ***	Supported
H6e: IOC → PEOU	0.324	6.662 ***	Supported
H6f: IOC → BI	0.109	2.376 *	Supported

Note: BI, behavioural intention; AT, attitude; PBC, perceived behavioural control; SN, subjective norms; PU, perceived usefulness; PEOU, perceived ease of use; IOC, impact of COVID-19; * *p* < 0.05; ** *p* < 0.01; *** *p* < 0.001.

## Data Availability

The data used in this study can be provided upon request.
